# Perspectives of primary healthcare nurses on cultural practices contributing to late antenatal booking in South Africa

**DOI:** 10.4102/hsag.v30i0.2997

**Published:** 2025-09-17

**Authors:** Sharon H. Maluleke-Ngomane, Thifhelimbilu I. Ramavhoya

**Affiliations:** 1 Department of Nursing, Faculty of Health Sciences, University of Johannesburg, Johannesburg, South Africa; 2 Department of Nursing Science, Faculty of Health Sciences, University of Pretoria, Pretoria, South Africa; 3 Department of Nursing, Faculty of Health Sciences, University of Limpopo, Polokwane, South Africa

**Keywords:** cultural practices, cultural competency, cultural inertia, late antenatal booking, traditional health practitioners, religious interventions, pregnancy, religious belief

## Abstract

**Background:**

Primary health care (PHC) nurses are crucial in reducing late antenatal booking and early detection of pregnancy-related conditions to enhance the best perinatal outcomes. Research confirms that early antenatal care is a key intervention to safe motherhood, aimed at preventing perinatal adverse events; however, cultural practices of pregnant women have a role to play in late antenatal booking.

**Aim:**

This study explored and discussed the cultural practices of antenatal clients that contribute to late antenatal booking as related by PHC nurses.

**Setting:**

This study was conducted at PHC facilities in Mpumalanga province, South Africa.

**Methods:**

Following qualitative phenomenological design, PHC nurses’ perceptions of cultural practices of antenatal clients contributing to delayed antenatal booking were explored and described. Purposeful sampling was followed to sample PHC nurses with three or more years of experience to respond to in-depth, open-ended questions. Colaizzi’s thematic data analysis was used to facilitate emerging themes and sub-themes. Measures of trustworthiness were ensured for this study.

**Results:**

Results show that PHC nurses believe that antenatal clients intentionally book late and use herbs and religious interventions. Furthermore, PHC nurses neglect the cultural practices of antenatal clients.

**Conclusion:**

Primary health care nurses may encourage trusting relationships by making cultural adjustments and increasing cultural competency, which may increase early antenatal booking, reduce the use of harmful interventions and improve positive perinatal outcomes.

**Contributions:**

This study contributed to an awareness of cultural inertia among PHC nurses, which may be averting clients from openness to health promotion provided at PHC facilities.

## Introduction

### Background

Globally, the maternal mortality ratio (MMR) was 223 per 100 000 live births in 2020, with over 287 000 maternal deaths reported (World Health Organization [WHO] 2023). This means that there are about 800 maternal fatalities per day, or one every 2 min, with an estimated 545 maternal deaths per 100 000 live births (WHO 2023). In 2020, Middle, Western and Eastern Africa had 61.3% maternal fatalities, which had a higher percentage of more than 10% (perinatal mortality) fatalities among women of reproductive age because of maternal causes (WHO 2023). As such, WHO (2023) cited interdependent superdeterminants of negative perinatal outcomes, including the biological traits of humans; political, economic and cultural foundations of societies; and cultural beliefs surrounding pregnancy and childbirth. The superdeterminants are an indication that positive perinatal care is not an easy task that can only be done by one person, but all need to be involved, from the client to spouses, families, communities, the religious sector and the health sector (Schoppe–Sullivan & Fagan 2020). As such, the PHC system plays a pivotal role in linking the communities to a health system aimed at quality comprehensive care, inclusive of early antenatal care services, aiming at increasing access to early antenatal care. To increase access to maternal health care services, including early antenatal care, government health-related policies and initiatives that address the health needs of women and children were initiated with the aim of meeting Sustainable Development Goal 3 (SDG 3), which is good health and well-being for all (Sdg 2019). Regardless, antenatal clients present up late after 12 weeks of missed menstrual period, which creates a missed opportunity for early problem identification even when facilities are within reach.

A Global Study cites cultural factors as reasons for late antenatal booking. The study found that gender disparities, financial limitations, social support gaps and cultural norms were important social obstacles to early antenatal booking (Jamil, Majeed & Zafar 2024). Another study identified a number of significant social barriers to prenatal treatment, including experiences with stigma and prejudice, lack of understanding, transportation difficulties and traditional behaviours (Bico 2024). A healthy baby and a healthy mother represent cherished aspirations for families across all cultural backgrounds, as stated by (May 2024). Although the national health goals in many countries around the world support families in prioritising mothers and infants, maternal mortality and morbidity are on the rise, and some are influenced by cultural practices and beliefs. In recent international studies, there has not been much focus on cultural inclusion and equitable concerns. Although studies have shown a high correlation between poor pregnancy outcomes and minority ethnicity and deprivation, there has been a notable absence of ethnic and socio-economic variety among participants. A study conducted by Berry, Yadav and Hole (2024) showed that in other sectors, there is a concern that well-meaning excitement for implementing a post-pandemic vision of remote prenatal care might have unforeseen effects on quality and safety, including the reinforcement and exacerbation of existing inequalities. These results affirm the need for a hybrid and/or collaborative model of antenatal care with the aim of creating equitable quality care across all cultures, ethnic groups and socio-economic levels (Ducroisy & Demba 2024).

According to Vedam et al. (2024), the concept of cultural safety and cultural competence in health care emerged from research that found demands in the 1980s and 1990s for reparations of past and ongoing maltreatment of clients as pregnant women had endured and were still experiencing while receiving care. This understanding calls for cultural competence for PHC nurses who are at the first level of care for antenatal clients who seek early antenatal care. In this study, PHC nurses are nurses who are trained to provide PHC clinical skills at the point of entry into the health system, including early antenatal care services. The definition of cultural competence is a paradigm that places a strong emphasis on abilities, cross-cultural understanding and the individual level of care (Vedam et al. 2024). While cultural competency and cultural awareness are two aspects of culturally aware health frameworks that are aligned with cultural safety, there are also some significant differences between the two. A collection of congruent behaviors, attitudes, and policies forms a cohesive unit within a system, organisation, or among professionals, which enables them to function effectively in cross-cultural contexts (Vedam et al. 2024).

A similar approach was suggested in sub-Saharan Africa by a study conducted by Musie et al. (2024), whose results show that the health care system does not currently have clear guidelines or much coordination among practitioners when it comes to integrating cultural interventions related to pregnancy care, which is one of the challenges. In addition, one of the challenges facing rural communities in South Africa is the passive framework to enable collaboration between Western science and cultural-related interventions in maternal and child health care. Mzimbiri et al. (2024) confirmed a common recognition that differences in access to high-quality early antenatal health care services cause high rates of maternal mortality in various regions of the world. These findings justify the abnormally high MMR, accounting for around 70% of all maternal fatalities in sub-Saharan Africa. As a result, taking action to prevent maternal fatalities is essential, as it is the understanding of the variables affecting access to care. As such, this study aimed to explore the cultural practices of antenatal clients contributing to late antenatal booking as related by PHC specialists. The results of the study may provide a base for cultural competence for PHC nurses as the first point of contact of care for antenatal clients.

## Study purpose

The purpose of this study was to explore and describe the cultural practices of antenatal clients contributing to late antenatal booking as related by PHC nurses in the context of Ehlanzeni District in South Africa.

## Research methods and design

### Study designs

A qualitative exploratory descriptive and contextual design was used to facilitate the exploration and description of cultural practices of antenatal clients contributing to late antenatal booking as described by PHC nurses in the context of Mpumalanga in South Africa. In-depth, unstructured interviews were posed to individual PHC nurses using open-ended questions. Data were analysed using Colaizzi’s thematic analysis to discover meaning through themes and sub-themes to uncover new ideas from responses that were captured verbatim from participants during interviews (Colaizzi 1978).

### Study methods

The descriptive phenomenological qualitative method was used to allow the exploration of new ideas related to the phenomenon under research (Tukisi 2024), using descriptive methods to discover the meaning and portray a clear picture of the phenomenon under study (Hesse-Biber 2012). The paradigmatic point of view used in this study was interpretivism, supported by the constructivist theory.

### The study context

In order to gain a deeper understanding of the practices of antenatal clients contributing to late antenatal booking as related by PHC nurses caring for antenatal clients, the study was carried out in an authentic setting of PHC facilities where antenatal care services are rendered. The research was conducted in 5 out of 40 24-h PHC facilities providing basic antenatal care services, inclusive of PHC gateway facilities and mobile units of the deep rural district in Mpumalanga province. Each facility has an average of 12 PHC nurses who provide antenatal services to antenatal clients.

### Population and sampling methods

Following the University of Pretoria’s approval for the study, permission was also granted by Mpumalanga province to proceed with the research study. A rural sub-district was randomly chosen to take part in the study. Prior to the commencement of the research, additional consent was obtained from the operational managers (OMs) of the selected facilities who played a pivotal role in aiding the researcher with participant recruitment. To recruit participants, a convenient purposive sampling method was employed. Using personal knowledge, experience and judgement to sample the targeted sample involves choosing ‘typical’ or ‘representative’ units from the population to create samples. The proposed purpose sampling framework integrates the concepts of adaptability, inductive reasoning and coherence found within this qualitative research. It acts as a resource for novice researchers and postgraduate students, in addition to functioning as an assessment tool for academics, principal investigators and reviewers of journals (Ahmad & Wilkins 2024).

The OMs of each PHC facility played a crucial role in aiding the researcher with participant recruitment by coordinating potential participants to attend the information session so that they participate with understanding. Primary care nurses working at PHC facilities were selected for this study based on their expertise in providing antenatal care services and their capacity to deliver precise and comprehensive information about their antenatal clients. Full-time registered PHC nurses who have been providing antenatal services to antenatal clients for more than a year, who have consented to participate, as well as PHC nurses who have a midwifery qualification, or PHC nurses who have a midwifery qualification or an advanced midwifery working in this facility were included in the study. Primary care specialists voluntarily signed a written consent and were on duty on the day of the interview to have interviews conducted in a natural setting that was convenient to them. The intention was to include a PHC specialist who would provide rich data on the cultural practices of antenatal clients contributing to late antenatal booking. Recruitment of participants continued, and the study reached 13 participants. The sample size of the study was determined by saturation, which was reached at the 13th participant (Tight 2024).

### Inclusion criteria and exclusion criteria

#### Inclusion criteria

The inclusion criteria were full-time registered PHC nurses with 3 or more years of experience who have been providing antenatal services to antenatal clients in a PHC setting, have a midwifery qualification and voluntarily signed a consent form to participate in this study.

#### Exclusion criteria

The exclusion criteria were primary care nurses with 3 or more years of experience working in PHC facilities who refuse to sign a consent form to participate in the study and a PHC nurse who was not midwifery trained and has less than 3 years of experience.

### Recruitment process

Upon obtaining approval and authorisation from Mpumalanga province, further permission was sought from the OMs responsible for selected PHC facilities. The researcher organised a meeting with OMs who assisted in organising a meeting for an introductory session with all sampled participants who met the inclusion criteria. After this initial meeting, the researcher suggested face-to-face interactions at a time that was convenient to the participants, such as upon their arrival in the morning or during lunch breaks. In these interactions, the researcher clarified the study’s aims and objectives, and provided a concise overview of the research methodology to be employed. Interested participants were encouraged to participate in the study and were encouraged to ask clarity-seeking questions, which were addressed transparently. The researcher had a research assistant who assisted in the distribution of information letters that included contact information, reassuring participants that they could reach out at any time for further clarification on any issues that may arise post-contact sessions. A weekly visit was conducted during which the researcher invited volunteers to complete the necessary consent forms and, where possible, arrange dates, times and locations according to their preferences. The researcher stressed the fact that participation is entirely voluntary, and individuals may withdraw from the study at any time without any consequences. Recruitment of sampled participants continued until comprehensive data collection was achieved and data saturation was reached.

### Data gathering methods and processes

Face-to-face in-depth interviews were conducted in the board room, which was a natural setting of the facility where the OM conveniently prepared for private and confidential individual interviews. The researcher applied an unstructured interview using an open-ended question that generated an in-depth discussion between the researcher and the participant using an in-depth exploration of the perceptions of PHC nurses about cultural practices of antenatal clients contributing to late antenatal booking. The interviews were conducted in English; however, participants were encouraged to speak in their preferred language. The central question given was as follows: ‘What are cultural practices contributing to late antenatal booking?’ Probing was used to gain more information and clarity from the responses of each participant. English was used during the interviews with participants, though the researcher allowed participants to use their language so that they would be able to express themselves freely. As participants came into the interview room, demographic data were asked first, then followed by the main research question to allow a flow of information as described by the participant to avoid interruption.

The following research question guided the study: *What are the cultural practices of antenatal clients contributing to late antenatal booking?*

During data collection, field notes were captured by the research assistant, and interview data were audio recorded to ensure that all information gathered from participants was captured verbatim. The assistant researcher actively captured responses as the researcher engaged participants for 45 min to 60 min based on how participants responded to the questions. The researcher engaged in answering questions. Data were collected until the eleventh participant gave similar responses given by the previous participants, and the researcher determined that data saturation was reached. However, two more participants were added to the interviews to confirm the saturation, which gave a total of 13 participants. Data were collected for the period of 8 weeks from November to December 2023.

### Data analysis

Data were analysed using the thematic method of data analysis, where data collection and analysis were conducted simultaneously. Inductive thematic analysis was employed by the researcher following the seven thematic steps, according to (Colaizzi 1978). This type of analysis is described as a linear, seven-phase procedure that includes the following: (1) transcribing all the participants’ descriptions verbatim, (2) extracting significant statements, (3) creating formulated meanings, (4) building themes, (5) developing an exhaustive description, (6) identifying the fundamental structure of the phenomenon and (7) returning to participants for validation. In data analysis, the researcher transcribed all interviews verbatim with participants and read through each transcript several times to highlight each statement with a common meaning. The data analysis was conducted by the researcher and sent to the independent coder, who signed a confidentiality agreement. After carefully examining the data, the researcher and the independent coder came to a consensus about the grouping and development of themes that emerged from the data to give it meaning. The researcher utilised the report produced by the analysis to compare it with the available literature and look for any parallels or inconsistencies. The data analysis method was followed by literature control in order to assess the calibre of the data gathered in literature control documents of what is known about the subject by combining primary, secondary and tertiary sources.

### Trustworthiness

Trustworthiness in qualitative research was attained by implementing strategies that ensure credibility, dependability, conformability and transferability, as described by (Lincoln & Guba 1985). In ensuring credibility, the researcher had a prolonged engagement with participants and the data; triangulated sources; engaged in peer debriefing, member checking and persistent observation; ensured referential adequacy; and maintained structural coherence. Dependability was ensured by triangulation, stepwise replication, keeping an audit trail and using a code-recode strategy. To ensure conformability, the researcher maintained transcripts and provided an audit trail when needed to maintain neutrality. Transferability involves the provision of a detailed description of the target population as well as a dense description of the research methodology, nominated sample, and detailed and rich information about the study findings (Lincoln & Guba 1985). In this study, transferability was ensured by sampling the PHC nurses who are fully employed in the PHC setting and have years of experience working in the facility, which can be generalised to another similar setting. The applied sampling method and the population of the study render the study comparable if it had to be applied in another similar setting.

### Ethical considerations

Ethical principles were adhered to as participants were humans who had the right to safety, respect and anonymity. Furthermore, this study was conducted in accordance with the Helsinki Declaration as revised in 2013 and the *Protection of Personal Information Act* (POPIA). A confidentiality agreement was signed with the co-coder to ensure that information is managed in confidentiality. Pseudo names were used in the form of codes to replace participants’ names and ensure anonymity. The unstructured interviews took place in a private environment to ensure privacy. The University of Pretoria Higher Degree Ethics Committee granted the researcher permission to perform the study (ethical clearance no: 45/2021). Participants voluntarily signed written informed consent forms and confidentiality forms that are ethically required to safeguard the participants.

## Results

The PHC nurses who participated in this study, as indicated in Table 1, were coming from different ethnic groups, and all were female nurses of different age groups providing antenatal services in selected facilities. Among the 13 PHC nurses who participated in the study, 2 (15%) nurses were aged between 21 years and 30 years. Among these PHC specialists, 8 (62%) nurses were aged between 31 years and 40 years and 3 (23%) nurses were aged between 41 years and 45 years.

**TABLE 1:** Demographic data for primary health care nurses (*N* = 13).

Criteria	Characteristics	Frequency	Percentage
Age (years)	21–30	2	15
	31–40	8	62
	41–45	3	23
Qualifications	Midwifery	7	52
	Degree	4	32
	Master’s	2	16
Ethnic group	Swazi	3	23
	Mapulana	2	15
	Shangaan	8	61

*Source:* S.H.M.-N.

Two main themes emerged from this study. However, one main theme with four sub-themes will be discussed, as indicated in Table 2.

**TABLE 2:** Themes and sub-themes from primary health care specialist.

Themes	Sub-themes
1. Client-related practices	1.1 Intentional late booking by pregnant women 1.2 Delay because of cultural beliefs 1.3 Delay because of spiritual beliefs
2. PHC specialist-related practices	2.1 PHC specialists prioritise Western medical practice over cultural practices

PHC, primary health care.

### Practices of women during pregnancy

The results of this study show that PHC nurses are aware that antenatal clients believe in cultural practices that lead to delayed antenatal booking. Participants in this study stated that antenatal clients show up late for the first antenatal booking because they use herbs and visit spiritual leaders before they come to the clinic for antenatal booking. These results are discussed under two themes and four sub-themes, which are part of the themes that emerged from the study.

### Theme 1: Client-related practices

Three sub-themes emerged from this theme, as indicated in Table 2. Primary care specialists reported that pregnant women were intentionally booking late, though they were aware of the need to start antenatal care early. They further reported that pregnant women visited their traditional healers and prophets before they could use PHC services.

#### Sub-theme 1.1: Intentional late booking by pregnant women

The results of the study confirm that pregnant women typically do not attend their early antenatal appointments even if pregnancy can be detected early. The study found that even after receiving health education, pregnant women show up late for antenatal booking. One PHC nurse says that she is aware that the antenatal clients are from different backgrounds, but she is not able to differentiate which culture belongs to which ethnic group. However, she observed that all these clients have some element of cultural beliefs that guide their pregnancy care. The following participant quotes attest that antenatal clients intentionally show up late for antenatal care:

‘In this catchment area, there are Shangaans, Bapulanas, and Swazis, all of which are cultural people. I just do not the differences across the practice, but I have observed by now that they are mostly traditional, and there is a tendency of starting antenatal care late regardless of their awareness of their pregnancy.’ (P1, Female, Shangaan, 38 years old)

‘These antenatal clients like believing in cultural interventions, but they delay mostly because they are still taking their herbs from traditional help.’ (P3, Female, Shangaan, 41 years old)

‘Pregnant women won’t come to the clinic for early booking; they rather come late for unknown reasons.’ (P8, Female, Shangaan, 44 years old)

‘I worked for the department for 13 years now, but I have seen a few Antenatal clients booking early in this clinic.’ (P5, Female, Mupalana, 45 years old)

‘Even as we give health education, pregnant women always book late for no apparent reason.’ (P11, Male, Shangaan, 41 years old)

‘Pregnant women usually do not show up early for antenatal booking; even when working in another facility, I have not seen women booking early for antenatal care.’ (P7, Female, Swazi, 37 years old)

Participants stated that it is known that antenatal clients will present late for early antenatal clinic for several reasons. Others reported that they have 13 years of working with the Department of Health in a PHC setting, and they have not seen a lot of antenatal clients showing up for early antenatal clinics since they started working in PHC facilities. The participant further stated that even after they receive health education, they still show up late for antenatal booking and that it is a general practice even in other facilities.

#### Sub-theme 1.2: Delay because of cultural beliefs

The use of herbs and cultural intervention by antenatal clients is known to PHC nurses. However, PHC nurses are unwilling to learn cultural treatment modalities. Participants stated that antenatal clients like to believe in cultural interventions, which makes them delay early antenatal booking because they want to start by finding traditional help from herbalists or spiritual interventions from religious leaders. The following quotes attest to the fact that antenatal clients used herbs during pregnancy:

‘These antenatal clients like believing in cultural interventions, but they delay mostly because they are still taking their herbs from traditional help.’ (P6, Female, Shangaan, 43 years old)

‘These women believe a lot of traditional medicines; hence, they always drink mixtures that they get from traditionalists, that’s why they delay booking.’ (P9, Female, Swazi, 39 years)

‘Antenatal clients delay themselves because they like listening to older people teaching them about the cultural thing. Encouraging the use of herbs as soon as they realize they are pregnant, only to present themselves late at the clinic for the first antenatal booking.’ (P10, Female, Swazi, 38 years old)

Participants stated that antenatal clients follow cultural interventions early in the pregnancy. Participants reported that antenatal clients listen to older people teaching them about cultural issues; hence, they present late at the facility. Some participants stated that antenatal clients use herbs early in pregnancy and only come to the clinic late.

#### Sub-theme 1.3: Delay because of spiritual beliefs

Participants reported that pregnant women visit their Prophets before coming to antenatal care, which caused late antenatal care booking. One participant responded during an interview by saying they do not know the antenatal clients because they are churchgoers who believe in traditional treatments. Furthermore, PHC specialists are not ready to spend time on spiritual interventions. Another participant confirmed that antenatal clients consult prophets to get holy water or even rough salt to protect their pregnancy:

‘You do not know these people. They like following false prophets who are delaying their antenatal booking. I think so because a lot of them are either churchgoers or believe in traditional treatments, which I personally will not waste my time on.’ (P4, Male, Shangaan, 41 years old)

‘Antenatal clients don’t learn. They like to consult prophets who usually use mixed solutions to protect pregnancy. But those things become risky to pregnancy, and women end up with miscarriages.’ (P5, Female, Shangaan, 45 years old)

‘Yes, they consult prophets to get holy water or even rough salt to protect their pregnancy, and this is what is delaying them from coming to the clinic.’ (P13, Female, Shangaan, 28 years old)

The results from theme one show that PHC nurses have some extent of knowledge that antenatal clients engage in cultural practices that delay them from booking early before 12 weeks of gestation. Primary health care nurses verbalise that antenatal clients intentionally book late for antenatal care.

### Theme 2: Primary health care specialist-related practices

This theme discusses health care specialist-related factors that influence late antenatal booking. From this main theme, one sub-theme emerged as indicated in Table 2. Participants confirm that they are Western health intervention inclined, and they will not have time to understand and/or accommodate cultural practices.

#### Sub-theme 2.1: Primary health care specialists prioritise Western medical practice over cultural practices

The results of this study show that PHC nurses are not fully aware of their catchment populations. They do not know the different ethnic groups. However, they know that antenatal clients believe in cultural practices in their catchment area. Furthermore, they do not know the difference between the different cultures practised in their catchment population. The voices of the PHC nurses as follows confirm that the catchment areas are populated by cultural communities. Participants in this study stated that they know the communities in the catchment area. They know that there are Shangaans, Bapulana and Ba-Swazi, who are known to be cultural people. The participants indicated that even if they know the communities, they do not know their differences across all cultures. However, they know that these are cultural people. In addition, participants reported that there is no way they can follow the beliefs of cultural antenatal clients, which means the antenatal clients should take what the facilities offer, which will save their lives and that of their babies:

‘In my experience with this community, I learned to know them as traditional people. We taught them to stop these traditional practices, but they persist. The fact is that we cannot follow their traditional practices.’ (P12, Female, Shangaan, 40 years)

‘We use national guidelines to ensure the provision of quality antenatal care; we cannot use cultural practices or religious practices.’ (P9, Female, Mapulana, 39 years)

Participants stated that in their experience working with this community, they learned to know that their clients are traditional people; however, they do not know how to differentiate these cultures. The participants concluded by saying that they are not ready to accommodate any traditional practice as part of antenatal care because even if they try to discourage the practice, they do not succeed. Another participant said that they use national guidelines to ensure quality of care. She was adamant that they will not use cultural practices in their antenatal services.

## Discussion

### Client-related factors

Based on the responses of sub-themes one and two, PHC nurses know that antenatal clients engage in some cultural practices related to pregnancy care. However, they cannot point out exactly what it is that antenatal clients practice while they are delaying their first antenatal booking before 12 weeks of missed menstrual period. These results are supported by Howes (2024), who cited that there is a lack of dedicated care for women because of a lack of sensitivity to the needs of women and non-stigmatising language, supporting the continuity of interpersonal relations. In an African study, Lunda, Minnie and Lubbe (2024) reported that the facilitation of the ‘Respectful Maternity Care’ interface between women and service providers in terms of practice, language, behaviour and attitude would, therefore, ensure positive perinatal outcomes. Another study in Kenya supports this perception and confirms that pregnant women undervalued early antenatal care because they believed that being pregnant was a healthy condition or that it was not a fundamental problem that called for medical attention. In addition, prior healthy pregnancies for which women did not seek care demotivated them from starting antenatal care at an early stage (Muthoni 2023). Another study supported this phenomenon in that it found that antenatal clients showed up late for first antenatal care for no known reason, and most of them presented with urinary tract infections and sexually transmitted infections (Edyedu et al. 2025). This finding is supported by Thompson (2024), whose study confirmed that challenges such as cost, distance, cultural beliefs and others play a significant role in early antenatal booking; however, this study only explored cultural practices-related factors.

These are the words of PHC nurses who confirm that health education is offered at the clinic; however, antenatal clients continue to show up late for antenatal care. This finding is supported by a study conducted in Palestine, which found that pregnant women believed that first-trimester antenatal care appointments were solely for those who were pregnant and had certain illnesses, such as backaches, headaches and HIV and AIDS. Pregnancy in the first trimester was unrecognised by the women who believed it to be too early and unimportant to warrant obtaining antenatal care. The majority of people who began antenatal care in the first trimester had already dealt with illnesses and difficulties, such as past caesarean sections and abortions (Barak et al. 2023).

This study shows that antenatal clients use herbs to strengthen their pregnancy rather than come for early antenatal visits. A study conducted in Brazil by Souza et al. (2023) confirms that out of 811 clients recruited, 69 (8.5%) of them admitted to making tea from plant materials and drinking before they presented at the clinic. In an international study, the use of herbal treatment was confirmed at a prevalence of 22.6% (61/270), whereas the use of herbal remedies to treat maternal conditions was 90.2% (55/61). Only 24.6% (15/61) of the women who were expectant mothers received herbal remedies. Regarding attitudes towards herbal medicine use, about two-thirds of expectant women had previously used herbal medicines and believed them to be successful (Bouqoufi et al. 2023). Buor, Agyemang and Awuku (2023) supported these findings in their study that shows more than 82.0% of respondents had ever used herbal medicine while pregnant, and a significant portion of their medication came from herbalists. They frequently used ginger and neem tree leaves, and their main health concerns or issues during pregnancy were anaemia, malaria and stomach aches. Income and religion both showed a statistically significant correlation with the usage of herbal medication. These findings concur with a study conducted in Korea that confirmed that antenatal clients use herbs early in their pregnancy, which might be the contributing factor to late antenatal booking (Im et al. 2024). Herbs are used following the confirmation of pregnancy, which they use before they present themselves at the PHC facility, delaying their first antenatal visit before 12 weeks of missed menstrual period.

In a study conducted in the Middle East, 70 (41.2%) participants reported using herbal medicines during pregnancy, 52 (30.6%) participants reported using herbal medicines during childbirth and 82 (48.2%) participants reported using herbal medicines after delivery. Mint (33.5%) was the most commonly used herb throughout pregnancy, followed by ginger (27.6%), while cinnamon (18.2%) was used during childbirth, and fenugreek and ginger (34.1% and 25.3%) were used following delivery. The primary reason for using herbs during pregnancy was to maintain good health (24.0%) (Al-Johani et al. 2023). These results are confirmed in another study where the archipelago, the Philippines, showed a wide variety of cultures. The Philippines’ traditional healing methods and expertise are passed down from generation to generation and are based on the cultural beliefs of the indigenous people. As medicinal plants are less expensive and more frequently available than synthetic medications, people who live in rural areas have been using herbs to treat illnesses as a first-line intervention (Magtalas et al. 2023).

From this study, PHC nurses confirm that antenatal clients rely mostly on cultural interventions, which they assume is the contributory factor for the delay. This is heard from their statement from the interviews. Dako-Gyeke et al. (2023) supported this finding in their study that found antenatal clients confirming their belief in different cultures according to their ethnic groups, for example, not being allowed to crossroads in the first trimester, which influences delayed first antenatal visits. In another study, the welfare of the infant was found historically reliant on the herbal foods consumed by their mothers. For the sake of their children’s health, moms reportedly avoided eating liquids, spicy foods and green leafy vegetables. Experiences include weight loss, nipple irritation and backaches that moms related to excessive latching and breastfeeding when seated for an extended period of time. This finding is supported by a study conducted in Sierra Leone that demonstrated that cultural influences significantly affect the vast array of ethnic and religious beliefs that exist in sub-Saharan Africa.

Most cultural behaviours and attitudes have a significant impact on whether expectant moms seek medical attention early, before 12 weeks of confirmed pregnancy (Timbo et al. 2023).

The result of the study shows that PHC nurses are aware that antenatal clients use spiritual interventions before they come for their first antenatal visit, which may be contributing to delayed antenatal booking. Furthermore, antenatal clients are said to be consulting spiritual leaders who give the mixtures, holy water and/or salt to use to protect their pregnancy, which is delaying them from showing up for early antenatal booking. A separate study corroborates these findings, indicating that an understanding of cultural practices enhances awareness of how antenatal clients respond to their health needs. Furthermore, the study affirms that culture significantly influences the behaviours exhibited by antenatal clients in response to their health requirements (Singla et al. 2022).

Maina (2024) supported these findings, confirming that people’s perceptions of a health issue are influenced by their religious background. Because of increased sensitivity around religious beliefs, patients were particularly vocal about their expectations for maternity care and cervical screening. These exchanges needed to be delicate, but the research notes that patients weren’t always cordial because the nurses’ work was routine and felt like a conveyor belt to them that is programmed to deliver the same services for all despite their individual beliefs (Ojo-Aromokudu et al. 2023). Maharaj, Mohammadnezhad and Khan (2022) supported the understanding of PHC nurses as articulated in their study, confirming that PHC specialists need to have customer service training in order to treat patients with respect and not stereotype them to whom they consult when they learn that they are pregnant. Antenatal clients should have the freedom to choose what is best for their personal health as well as the health of their unborn child. The results of a study by Maharaj et al. (2022) indicated that antenatal clients like using prophets to address their health needs and tell them the outcome of their pregnancy at the beginning of pregnancy. Another study conducted in Pakistan supports this study’s findings in that antenatal clients still encounter difficulties and hindrances while trying to attain equal opportunities in the health sector (Iqbal & Majeed 2023).

### Primary health care specialist-related practices

In this study, nurses confirm that they focus on the use of the policies and guidelines and not cultural or religious practices in pregnancy care. A study by AlDughaishi, Matua and Seshan (2023) supported this finding in that their study found that PHC nurses gave health education to clients according to the maternal guidelines and did not consider the client’s knowledge. Furthermore, PHC nurses offer education to antenatal clients on different aspects of antenatal care without giving them an opportunity to clients to share what they know and believe (AlDughaishi et al. 2023). It is evident in this study that barriers to antenatal care also emanate from PHC nurses’ perceptions and not from antenatal clients who experienced the phenomenon under research.

Mantula et al. (2023) confirmed that there is inefficiency of care when incoherent midwife–woman collaboration occurs when women’s cultural ideas are not considered while creating maternity care plans. The care given to women during labour and childbirth was judged to be inadequate in terms of emotional, physical and educational assistance. The implication is that PHC nurses need to offer woman-centred perinatal care and are not sensitive to cultural practices known to antenatal clients to be of value. The statement above confirms that PHC nurses are not ready to accommodate cultural and/or spiritual interventions that perinatal clients believe in. They are committed to use guidelines to provide antenatal care across all cultures and religions. This result is supported by another study that explains that despite doctors’ best attempts to deliver care in resource-constrained circumstances, women’s Afari ethnicity has a significant impact on the level of clinical tensions, access to cultural safety and injustices they have or do not have (Hagaman et al. 2023). Furthermore, a study conducted in South Africa found that obstacles such as organisational culture and the personally adapted views of the PHC nurses hinder them from honouring the culture of the child-bearing age women, preventing the PHC specialists from providing culturally appropriate care. These results are supported by Alatinga, Affah and Abiiro (2021) in a study, which confirmed that PHC nurses know that pregnant women even deliver each other at home to create an opportunity to apply cultural interventions, and nurses are not prepared to reach out to understand these cultural practices because they rely on national guidelines to provide care to antenatal clients.

A strong political will, through a wide consultative framework and investment in research, will enhance understanding of how to create compassionate and accessible health service points. Most importantly, it requires leaders and policymakers who understand the value and importance of bringing ‘compassion’ back to maternity workplace culture. Evidence from the study reports that PHC nurses view antenatal clients as visiting traditional healers and spiritual leaders as soon as they miss their menstrual period. However, they would not visit the PHC facility. It is recommended that when caring for women during the peripartum period, medical professionals should routinely record the history of common traditional practices to help them deliver high-quality care by eliminating all harmful customs and supports (Musie et al. 2024).

### Implications and recommendations

This study has implications for policymakers on strategies to improve early antenatal booking at PHC facilities, which is the first point of contact for antenatal clients. Primary health care nurses need to take note of the antenatal client’s cultural beliefs and reach out early to prevent late booking. It is therefore recommended that PHC nurses should engage antenatal clients to understand their needs and empower them based on their beliefs. Including PHC nurses in client-centred cultural needs could also advance pathways to collaborative PHC services to support the antenatal clients’ access to comprehensive early antenatal care. Cultural and religious practices must be addressed in the PHC curriculum to prepare PHC nurses to be culturally competent. Inservice training should be offered to PHC nurses to help them embrace the beliefs, values, morals and world views of the antenatal clients, which may encourage them to show up early at the facility for early antenatal booking. By addressing the socio-cultural practice, traditional leaders could draft laws with the help of PHC specialists to encourage pregnant clients to come for early antenatal care. A greater understanding of the timing and significance of early antenatal care booking is required.

## Conclusion

The findings of this study reveal that PHC nurses know that antenatal clients have beliefs and practices that they do immediately when they miss a menstrual period; however, they are not ready to explore these practices because their curriculum does not include cultural beliefs. This phenomenon encourages distinctions between the client and their PHC nurses, which may perpetuate a relationship of mistrust and lack of partnership between the antenatal client and the PHC nurse.

## References

[CIT0001] Ahmad, M. & Wilkins, S., 2024, ‘Purposive sampling in qualitative research: A framework for the entire journey’, *Quality & Quantity* 59, 1461–1479. 10.1007/s11135-024-02022-5

[CIT0002] Alatinga, K.A., Affah, J. & Abiiro, G.A., 2021, ‘Why do women attend antenatal care but give birth at home? A qualitative study in a rural Ghanaian District’, *PLoS One* 16(12), e0261316. 10.1371/journal.pone.0261316 34914793 PMC8675692

[CIT0003] AlDughaishi, M.Y., Matua, G.A. & Seshan, V., 2023, ‘A qualitative inquiry of women’s perspective of antenatal education services in Oman’, *SAGE Open Nursing* 9, 23779608231159336. 10.1177/23779608231159336 36895708 PMC9989448

[CIT0004] Al-Johani, L.E., AlGhamdi, K.S., AlShaary, A., Aljohani, M.E., Alolayan, A.B. & Elsayed, S.A., 2023, ‘Prevalence of herbal use for obstetrics purposes in Almadinah Almunawwarah, Saudi Arabia’, *Journal of Herbal Medicine* 42, 100756. 10.1016/j.hermed.2023.100756

[CIT0005] Barak, O., Lovelace, T., Piekos, S., Chu, T., Cao, Z., Sadovsky, E. et al., 2023, ‘Integrated unbiased multiomics defines disease-independent placental clusters in common obstetrical syndromes’, *BMC Medicine* 21(1), 349. 10.1186/s12916-023-03054-8 37679695 PMC10485945

[CIT0006] Berry, L.L., Yadav, M.S. & Hole, M.K., 2024, ‘Reclaiming healthcare’s healing mission for a sustainable future’, *Journal of Service Research* 27(1), 6–27. 10.1177/10946705231198024

[CIT0007] Bico, M.S., 2024, ‘Factors associated with delayed utilization of antenatal care services among pregnant women: A case study at Jinja Regional Referral Hospital, Uganda’, *IDOSR Journal of Biology, Chemistry and Pharmacy* 9(1), 29–36. 10.59298/IDOSR/JBCP/24/91.2936

[CIT0008] Bouqoufi, A., Laila, L., Boujraf, S., Hadj, F.A.E., Razine, R., Abouqal, R. et al., 2024, ‘Prevalence and associated factors of self-medication in worldwide pregnant women: Systematic review and meta-analysis’, *BMC Public Health* 24(1), 308. 10.1186/s12889-023-17195-1 38279083 PMC10821266

[CIT0009] Buor, D., Agyemang, S. & Awuku, P., 2023, ‘The determinants of utilisation of herbal medicine among pregnant women in the Asante Akim North District, Ghana’, *Maternal and Child Health Journal* 27(10), 1886–1896. 10.1007/s10995-023-03676-7 37209378

[CIT0010] Colaizzi, P.F., 1978, ‘Psychological research as the phenomenologist views it’, in R.S. Valle & M. King (eds.), *Existential-phenomenological alternatives for psychology*, p. 6, Oxford University Press, New York, NY.

[CIT0011] Dako-Gyeke, P., Hornuvo, R., Glozah, F.N., Asampong, E., Tabong, P.T.-N., Nwameme, A. et al., 2023, ‘Pilot implementation of community health advocacy teams to improve the effectiveness of long-lasting insecticide net distribution through both campaigns and continuous channels in Ghana: A qualitative study of opportunities and barriers to implementation’, *Frontiers in Public Health* 11, 1133151. 10.3389/fpubh.2023.1133151 37583887 PMC10423875

[CIT0012] Ducroisy, A. & Demba, N., 2024, ‘Racial and ethnic disparities in Neuraxial analgesia use among laboring women: A systematic review’, Doctoral dissertation, Franciscan Missionaries of Our Lady University, viewed 17 February 2025, from https://search.proquest.com/openview/.

[CIT0013] Edyedu, I., Ugwu, O.P.C., Ugwu, C.N., Alum, E.U., Eze, V.H.U., Basajja, M. et al., 2025, ‘The role of pharmacological interventions in managing urological complications during pregnancy and childbirth: A review’, *Medicine* 104(7), e41381. 10.1097/MD.0000000000041381 39960970 PMC11835077

[CIT0014] Hagaman, A., Rodriguez, H.G., Egger, E., Bitewulign, B., Case, H., Alemayehu, A.K. et al., 2023, ‘Navigating and manipulating childbirth services in Afar, Ethiopia: A qualitative study of cultural safety in the birthing room’, *Social Science & Medicine* 331, 116073. 10.1016/j.socscimed.2023.116073 37481879 PMC10410251

[CIT0015] Hesse-Biber, S., 2012, ‘Weaving a multimethodology and mixed methods praxis into randomized control trials to enhance credibility’, *Qualitative Inquiry* 18(10), 876–889. 10.1177/1077800412456964

[CIT0016] Howes, E., 2024, ‘Indigenous health and healing in Otavalo, Ecuador: An interpretative phenomenological study’, Doctoral dissertation, The Chicago School of Professional Psychology, viewed 02 February 2025, from https://search.proquest.com/openview/.

[CIT0017] Im, H.B., Hwang, J.H., Choi, D., Choi, S.J. & Han, D., 2024, ‘Patient-physician communication on herbal medicine use during pregnancy: A systematic review and meta-analysis’, *BMJ Global Health* 9(3), e013412. 10.1136/bmjgh-2023-013412 PMC10916170 38448037

[CIT0018] Iqbal, K. & Majeed, D.H.A., 2023, ‘From Prophet Muhammad’s era to modern times: Empowering women in medical activities for a healthier Pakistan’, *INKISHAF* 3(8), 116–133, viewed 20 January 2025, from http://inkishaf.org/index.php/home/article/view/19.

[CIT0019] Jamil, S., Majeed, T. & Zafar, T., 2024, ‘Social barriers obstructing early antenatal care’, *Medical Forum Monthly* 35(2), 32–36. 10.60110/medforum.350207

[CIT0020] Lincoln, Y.S. & Guba, E.G., 1985, *Naturalistic inquiry*, Sage Publication, The International Publication, Newberry Park, CA.

[CIT0021] Lunda, P., Minnie, C.S. & Lubbe, W., 2024, ‘Perspectives of midwives on respectful maternity care’, *BMC Pregnancy and Childbirth* 24(1), 721, viewed n.d., from https://link.springer.com/content/pdf/10.1186/s12884-024-06894-1.pdf. 39506692 10.1186/s12884-024-06894-1 PMC11539527

[CIT0022] Magtalas, M.C., Balbin, P.T., Cruz, E.C., Guevarra, R.C., Cruz, A.R.D.P., Silverio, C.E. et al., 2023, ‘A systematic review of ethnomedicinal plants used for pregnancy, childbirth, and postpartum care in the Philippines’, *Phytomedicine Plus* 3(1), 100407. 10.1016/j.phyplu.2023.100407

[CIT0023] Maharaj, R., Mohammadnezhad, M. & Khan, S., 2022, ‘Characteristics and predictors of late antenatal booking among pregnant women in Fiji’, *Maternal and Child Health Journal* 26(8), 1667–1675. 10.1007/s10995-022-03443-0 35476167

[CIT0024] Maina, R., 2024, ‘Maternal antenatal care access and attendance: A qualitative study of midwives’ and pregnant women’s experiences in Kilifi, Kenya’, Doctoral dissertation, University of Salford, viewed 21 February 2024, from https://salford-repository.worktribe.com/preview/2370222/.

[CIT0025] Mantula, F., Chamisa, J.A., Nunu, W.N. & Nyanhongo, P.S., 2023, ‘Women’s perspectives on cultural sensitivity of midwives during intrapartum care at a maternity ward in a National Referral Hospital in Zimbabwe’, *SAGE Open Nursing* 9, 23779608231160476. 10.1177/23779608231160476 36875792 PMC9974627

[CIT0026] May, A., 2024, ‘The impact of antenatal health education on healthy pregnancy and safe delivery’, *Journal of Social Research* 3(3), 858–865. 10.55324/josr.v3i3.1970

[CIT0027] Musie, M.R., Mulaudzi, F.M., Anokwuru, R. & Sepeng, N.V., 2024, ‘An inclusive framework for collaboration between midwives and traditional birth attendants and optimising maternal and child healthcare in restricted rural communities in South Africa: Policy considerations’, *Healthcare* 12(3), 363. 10.3390/healthcare12030363 38338248 PMC10855344

[CIT0028] Muthoni, A.B.W., 2023, *Determinants of timely initiation of antenatal care among pregnant women attending antenatal clinic at Embu County Referral Hospital, Kenya*, viewed 22 October 2025, from http://ir.jkuat.ac.ke/bitstream/handle/.

[CIT0029] Mzimbiri, R.I., Msokwe, D. & Mike, A.F., 2024, ‘Socio-cultural factors influencing the utilization of antenatal health care services in Pageri administrative area, South Sudan’, *Open Access Library Journal* 11(5), 1–12. 10.4236/oalib.1111570

[CIT0030] Ojo-Aromokudu, O., Suffel, A., Bell, S. & Mounier-Jack, S., 2023, ‘Views and experiences of primary care among Black communities in the United Kingdom: A qualitative systematic review’, *Ethnicity & Health* 28(7), 1006–1025. 10.1080/13557858.2023.2208313 37160684

[CIT0031] Schoppe-Sullivan, S.J. & Fagan, J., 2020, ‘The evolution of fathering research in the 21st century: Persistent challenges, new directions’, *Journal of Marriage and Family* 82(1), 175–197, viewed n.d., from https://onlinelibrary.wiley.com/doi/pdf/10.1111/jomf.12645?casa_token=.

[CIT0032] Sdg, U., 2019, *Sustainable development goals. The energy progress report*, Tracking SDG, 7, pp. 805–814, viewed n.d., from https://www.mitelspa.it/wp-content/uploads/2023/09/Piano-Strategico-Mitel-Sostenibilita.pdf.

[CIT0033] Singla, D.R., Hossain, S., Andrejek, N., Cohen, M.J., Dennis, C.L., Kim, J. et al., 2022, ‘Culturally sensitive psychotherapy for perinatal women: A mixed methods study’, *Journal of Consulting and Clinical Psychology* 90(10), 770. 10.1037/ccp0000754 36174135

[CIT0034] Souza, C.D.S.M., Camargo, E.B., Riera, R., Lima, R.V., De Oliveira Andrade, E., Betrán, A.P. et al., 2023, ‘Use of medicinal herbs in natural by pregnant women in the Amazon region’, *Journal of Herbal Medicine* 41, 100691. 10.1016/j.hermed.2023.100691

[CIT0035] Thompson, O., 2024, ‘Beyond our pregnancies and bodies’: Benefits and barriers to antenatal care clinic utilisation among pregnant women in Abeokuta, southwest Nigeria’, *African Identities* 1–18. 10.1080/14725843.2024.2388180

[CIT0036] Tight, M., 2024, ‘Saturation: An overworked and misunderstood concept?’, *Qualitative Inquiry* 30(7), 577–583. 10.1177/10778004231183948

[CIT0037] Timbo, A., Fallah, J., Bourne, P.A. & Muchee, T., 2023, *The perceptions of healthcare workers on factors influencing infant mortality in the Western Area Rural District, Sierra Leone*, viewed 22 October 2024, from https://www.researchgate.net/profile/Paul-Bourne/publication/369196413.

[CIT0038] Tukisi, K.P., 2024, ‘Midwives’ descriptions of policies on access to maternity health services in Northwest Province, South Africa’, *International Journal of Research in Business & Social Science* 13(5), 960–971. 10.20525/ijrbs.v13i5.3503

[CIT0039] Vedam, S., Stoll, K., Tarasoff, L., Phillips-Beck, W., Lo, W., MacDonald, K. et al., 2024. ‘The RESPCCT study: Community-led development of a person-centered instrument to measure health equity in perinatal services’, *Journal of Participatory Research Methods* 5(1), viewed n.d., from https://jprm.scholasticahq.com/article/94399.pdf.

[CIT0040] World Health Organization (WHO), 2023, *WHO recommendations on antenatal care for a positive pregnancy experience: Screening, diagnosis, and treatment of tuberculosis disease in pregnant women. Evidence-to-action brief: Highlights and key messages from the World Health Organization’s 2016 global recommendations*, World Health Organization, viewed 21 October 2024, from https://books.google.com/books.

